# Salinity Tolerance in a Synthetic Allotetraploid Wheat (S^l^S^l^AA) Is Similar to Its Higher Tolerant Parent *Aegilops longissima* (S^l^S^l^) and Linked to Flavonoids Metabolism

**DOI:** 10.3389/fpls.2022.835498

**Published:** 2022-03-17

**Authors:** Tiansi Fu, Chenyang Xu, Hong Li, Xiaohan Wu, Man Tang, Binbin Xiao, Ruili Lv, Zhibin Zhang, Xiang Gao, Bao Liu, Chunwu Yang

**Affiliations:** Key Laboratory of Molecular Epigenetics of the Ministry of Education (MOE), Northeast Normal University, Changchun, China

**Keywords:** allopolyploidy, synthetic wheat, salinity tolerance, flavonoid, ROS, metabolome

## Abstract

Allotetraploidization between A and S (closely related to B) genome species led to the speciation of allotetraploid wheat (genome BBAA). However, the immediate metabolic outcomes and adaptive changes caused by the allotetraploidization event are poorly understood. Here, we investigated how allotetraploidization affected salinity tolerance using a synthetic allotetraploid wheat line (genome S^l^S^l^AA, labeled as 4x), its *Aegilops longissima* (genome S^l^S^l^, labeled as S^l^S^l^) and *Triticum urartu* (AA genome, labeled as AA) parents. We found that the degree of salinity tolerance of 4x was similar to its S^l^S^l^ parent, and both were substantially more tolerant to salinity stress than AA. This suggests that the S^l^S^l^ subgenome exerts a dominant effect for this trait in 4x. Compared with S^l^S^l^ and 4x, the salinity-stressed AA plants did not accumulate a higher concentration of Na^+^ in leaves, but showed severe membrane peroxidation and accumulated a higher concentration of ROS (H_2_O_2_ and O_2_^⋅⁣–^) and a lesser concentration of flavonoids, indicating that ROS metabolism plays a key role in saline sensitivity. Exogenous flavonoid application to roots of AA plants significantly relieved salinity-caused injury. Our results suggest that the higher accumulation of flavonoids in S^l^S^l^ may contribute to ROS scavenging and salinity tolerance, and these physiological properties were stably inherited by the nascent allotetraploid S^l^S^l^AA.

## Highlights

- *Triticum urartu* (AA genome) shows a clear defect in the synthesis/accumulation of many flavonoids.
- Flavonoids play a vital role in salinity tolerance differentiation among wheat lines with different genome components.
- Synthetic allotetraploid wheat (genome S^l^S^l^AA) acquires strong salinity tolerance by directly inheriting the flavonoid metabolism traits of its *Aegilops longissima* (S^l^S^l^) parent.

## Introduction

Polyploidy, or whole-genome duplication (WGD), is a driving force in the evolution and diversification of all organisms, especially predominant in higher plants (Fox et al., 2020); this is evidenced by the fact that all angiosperm species have undergone one or more WGD events during their evolutionary histories (Jiao et al., 2011; Soltis et al., 2015; Van de Peer et al., 2017; Fox et al., 2020). Taxonomically, polyploids are classified into two major types, autopolyploidy, i.e., WGD of a single species and allopolyploidy that invokes WGD of a hybrid of two or more species. Many important crops, such as wheat, *Brassica napus*, coffee, and cotton, are allopolyploids, suggesting greater responsiveness of doubled hybrid genomes to strong artificial selections under domestication. One unique biological property of allopolyploidy is the combination of advantageous traits of parental species (Comai, 2005; Chen, 2007). However, the phenotypic outcome of merging two or more divergent genomes into one nucleus is not straightforwardly predictable due to, among other factors, the complex interaction and rewiring of gene regulatory networks of distinct species (Yoo et al., 2014).

Plants produce a huge array of metabolites, far more than other organisms do (Fang et al., 2019). Biomolecules synthesized in plants, such as phytohormones, carbohydrates, lipids, nucleic acids, amino acids, vitamins, and some secondary metabolites, play essential roles in the growth, development, and adaptation of plants. These different types of metabolites coordinate to construct complex metabolism networks, in which a change of any metabolite may influence the synthesis of many other metabolites (Buchanan et al., 2015). Natural selection can lead to the extension or deletion of metabolic steps of a pathway *via* mutation of enzymes, resulting in the loss of metabolites or the emergence of new metabolites (Firn and Jones, 2004).

The *Triticum*–*Aegilops* complex contains single or combinations of 8 genomes (A, C, D, U, T, M, N, and S) (Zohary and Feldman, 1962; Zhang et al., 2016). Of these, only A-, S- (closely related to B), and D-genome species were involved as subgenome donors to hexaploid common wheat (*Triticum aestivum*, genome BBAADD). Common wheat is formed through two allopolyploidization events (Uauy, 2017). Allotetraploidization between A and S genomes led to the speciation of wild allotetraploid wheat (*Triticum turgidum*, genome BBAA) 0.5–0.8 million years ago (Huang et al., 2002; Dvorak and Akhunov, 2005; Gornicki et al., 2014; Marcussen et al., 2014). Common wheat was formed following the combination of BBAA genome from domesticated allotetraploid wheat and DD genome from *Aegilops tauschii via* allohexaploidization 8,500–10,000 years ago (Dubcovsky and Dvorak, 2007; Uauy, 2017). Although many studies have been conducted on the evolution of common wheat, less attention has been paid to the study of tetraploid wheat and its putative diploid progenitors.

The A- and S-genome diploid species of the *Triticum*–*Aegilops* complex are diverged from a common ancestor about 6.5 million years ago (Huang et al., 2002; Dvorak and Akhunov, 2005; Gornicki et al., 2014; Marcussen et al., 2014; Li et al., 2021). These species inhabit distinct ecological niches and should have evolved species-specific secondary metabolism pathways. It is therefore of interest to investigate the metabolic outcomes when the divergent genomes are combined by allotetraploidization. Indeed, several prior studies have reported that allopolyploidization influences growth and adaptability *via* mediating the accumulation of key metabolites (Lou and Baldwin, 2003; Pearse et al., 2006; Banyai et al., 2010; Xing et al., 2011). For example, innovation of herbivore resistance in synthetic allopolyploid *Nicotiana* X *mierata* was linked to changed concentrations of secondary metabolites (Pearse et al., 2006).

Soil salinization is a severe global environmental factor limiting crop production. Wheat is relatively salinity-tolerant compared with other food crops (Munns and Tester, 2008). It was found that synthetic hexaploid wheats (genome BBAADD) show immediately enhanced salinity tolerance following allohexaploidization (Yang et al., 2014); however, how allotetraploidization influences salinity tolerance is unclear. In this study, we focused on salinity tolerance changes in a synthetic allotetraploid wheat line (genome S^l^S^l^AA) formed by crossing *Aegilops longissima* (genome S^l^S^l^) and *Triticum urartu* (genome AA). We compared the physiological and metabolomic responses of this synthetic allotetraploid wheat line and its diploid parents under both normal and salinity stress conditions.

## Results

### Physiological Response

**FIGURE 1:**
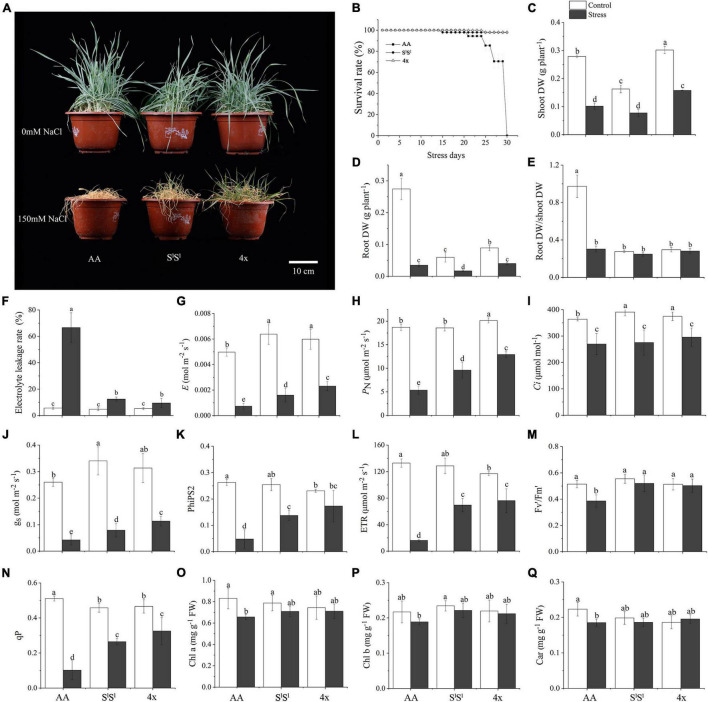
Effects of salinity stress on growth and photosynthesis in a synthetic tetraploid wheat line and its diploid parents. The synthetic tetraploid wheat line (genome S^l^S^l^AA, labeled as 4x) was generated by crossing and chromosome-doubling of *Triticum urartu* (AA genome, labeled as AA) and *Aegilops longissima* (genome S^l^S^l^, labeled as S^l^S^l^). The seedlings were exposed to 150 mM NaCl for 7 **(C–Q)** and 30 **(A,B)** days. The values are the mean (± SD) of three biological replicates. Different letters above the bar showed significant differences among wheat lines and treatments according to *t*-test (*P* < 0.05). Fv/Fm, maximum quantum efficiency of photosystem II; Fv′/Fm′, effective quantum efficiency of PSII; PhiPS2, real quantum efficiency of PSII; qP, photochemical quenching; *P*_*N*_, net photosynthetic rate, *g*_*s*_, stomatal conductance, Ci, intercellular CO2 concentration, *E*, transpiration rate; ETR, electron transport rate; Chl, chlorophyll; Car, carotenoid.

We used 4x, AA, and S^l^S^l^ to denote the synthetic tetraploid wheat line (S^l^S^l^AA), its diploid parents, *Triticum urartu* (AA), and *Aegilops longissima* (S^l^S^l^), respectively. We compared the salinity tolerance levels of the three lines and found that the S^l^S^l^ and 4x plants showed similar better growth and higher survival rate than AA plants under salinity stress (Figures 1A,B). Salinity stress increased electrolyte leakage rate in all three lines, but AA plants showed a much higher electrolyte leakage rate than did S^l^S^l^ and 4x plants under salinity stress. Salinity stress strongly reduced root dry weight (DW) in all three lines, but with a greater reduction in AA (87.3%) than in S^l^S^l^ (71.4%) and 4x (55.1%). However, salinity stress produced similar inhibiting effects on shoot growth of all three lines. In addition, salinity stress decreased the root DW/shoot DW ratio of AA plants but did not affect those of S^l^S^l^ and 4x (Figures 1C–F). Under salinity stress, net photosynthesis rate, transpiration rate, and stomatal conductance were much lower in AA than in 4x and S^l^S^l^ plants (Figures 1G–J). We measured chlorophyll fluorescence parameters as they reflect the status of photosynthetic electron transport (light reaction of photosynthesis). Values of all the three chlorophyll fluorescence parameters (PhiPS2, ETR, and qP) showed a greater reduction in AA than in S^l^S^l^ and 4x plants (Figures 1K–N). Salinity stress decreased Fv′/Fm′ in AA but not in S^l^S^l^ and 4x plants (Figure 1M). Salinity stress reduced the concentration of chlorophyll A and carotenoid in AA but not in S^l^S^l^ and 4x plants (Figures 1O–Q). Together, all the measured physiological parameters consistently point to similar salinity tolerance levels of 4x and its S^l^S^l^ parent, both of which are much higher than that of AA plants.

### Antioxidant Compounds

**FIGURE 2:**
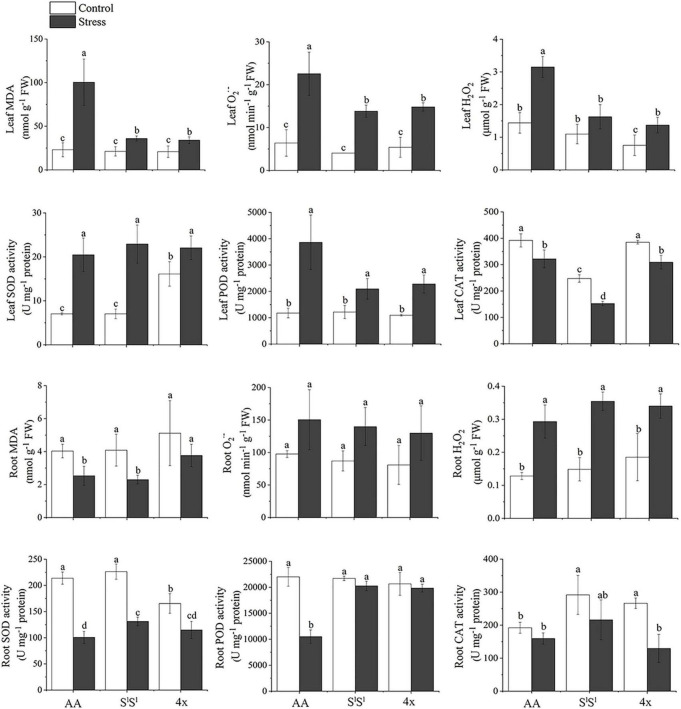
Effects of salinity stress on antioxidant systems of the synthetic tetraploid wheat and its diploid parents. The seedlings were exposed to 150 mM NaCl for 7 days. The values are mean (± SD) of three biological replicates. Different letters above bars denote significant differences among the lines and treatments according to *t*-test (*P* < 0.05).

H_2_O_2_ and superoxide anion radical (O_2_^⋅⁣–^) are two major reactive oxygen species (ROS). In all three lines, salinity stress enhanced H_2_O_2_ and O_2_^⋅⁣–^ concentrations in leaves but unaffected their accumulation in roots. In leaves, salinity stress-induced elevation in both H_2_O_2_ and O_2_^⋅⁣–^ concentrations were much greater in AA than in S^l^S^l^ and 4x plants (Figure 2). Under salinity stress, AA plants also showed much higher concentrations of H_2_O_2_ and O_2_^⋅-^ in leaves. The accumulation of H_2_O_2_ and O_2_^⋅⁣–^ in roots was not affected by salinity stress in all three lines. Malondialdehyde (MDA) reflects the production of peroxidation of ROS to the membrane. AA showed much higher MDA concentration in leaves than S^l^S^l^ and 4x under salinity stress (Figure 2), indicating that the damage of ROS to leaf membrane was much severe in AA than in S^l^S^l^ and 4x plants. In summary, under salinity stress, compared with S^l^S^l^ and S^l^S^l^AA, leaves of AA accumulated a higher concentration of ROS that may have caused a greater damage to the membrane system.

### Ion Contents

**FIGURE 3:**
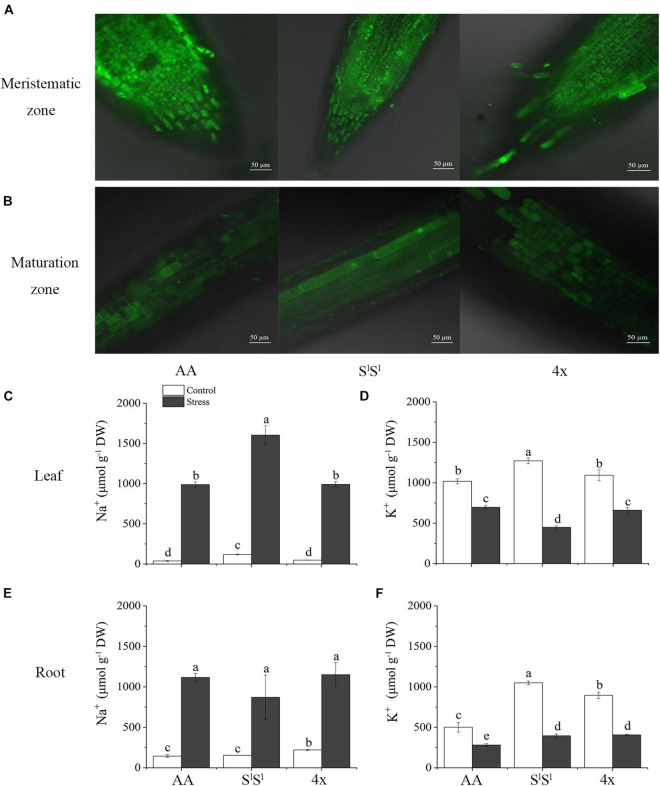
Effects of salinity stress on Na^+^ and K^+^ homeostasis in a synthetic tetraploid wheat line and its diploid parents. **(A,B)** Na^+^ distribution in root cell indicated by CoroNa Green AM under salinity stress. **(C–F)** Na^+^ and K^+^ concentration. The seedlings were exposed to 150 mM NaCl for 7 days. The values are the mean (± SD) of three biological replicates. Different letters above the bar showed significant differences among wheat lines and treatments according to *t*-test (*P* < 0.05).

We examined intracellular Na^+^ distribution in root cells with CoroNa Green AM under normal (Supplementary Figure 1) and salinity stress conditions (Figures 3A,B), and measured Na^+^ and K^+^ concentrations in leaves and roots of all the three lines (Figures 3C–F). Under salinity stress, AA did not display higher Na^+^ concentration and lower K concentration than S^l^S^l^ and 4x (Figures 3C–F). Actually, Na^+^ concentration was much higher in S^l^S^l^ than in 4x and AA leaves under salinity stress (Figure 3C). In roots, AA also did not show a higher Na^+^ concentration than S^l^S^l^ and 4x (Figure 3E). Also, confocal laser scanning microscopy with CoroNa Green AM revealed that AA did not show higher intracellular Na^+^ concentration than S^l^S^l^ and 4x in meristem cells and maturation zones of roots under salinity stress (Figures 3A,B and Supplementary Figure 2).

### Metabolome Profiling

**FIGURE 4:**
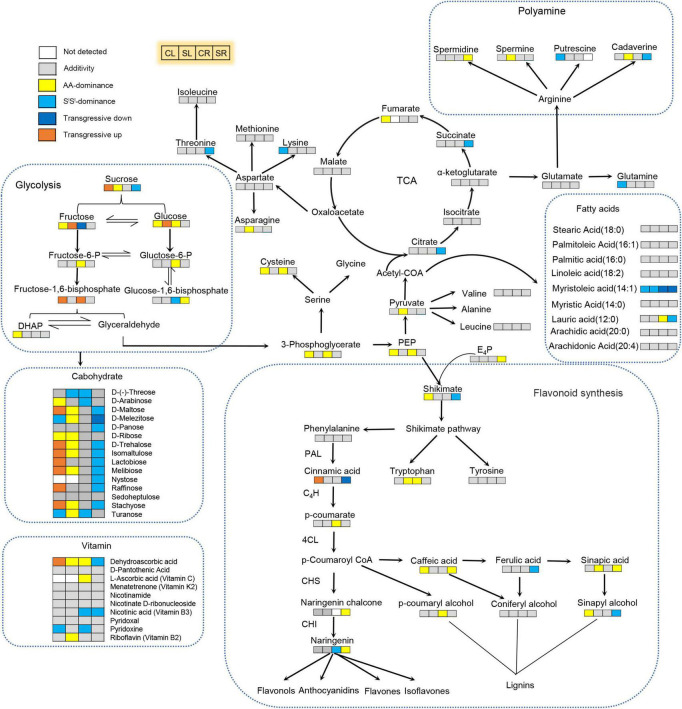
Integrative effects of salinity stress and allotetraploidization on metabolism in a synthetic tetraploid wheat line. Non-additive and additive accumulation of key metabolites in the synthetic tetraploid wheat line were marked on the primary metabolism network. The seedlings were exposed to 150 mM NaCl for 7 days. CL, control leaf; SL, stress leaf; CR, control root; SR, stress root. Joined four rectangle boxes denoted control leaf (CL), stress leaf (SL), control root (CR), and stress root (SR), respectively.

Collectively, we detected 895 metabolites in leaves of the three lines, which included 72 alkaloids, 68 free fatty acids, 208 flavonoids, 87 amino acids and derivatives, 84 organic acids, 52 nucleotides and derivatives, 49 saccharides or alcohols, 14 vitamins, 119 phenolic acids, 35 lignans or coumarins, 7 terpenoids and 100 others (Supplementary Tables 1, 2 and Supplementary Figure 3A). These metabolites covered key metabolites of almost all metabolism pathways (Figure 4). Of the 895 metabolites, 14 (10 flavonoids, 2 lignans or coumarins, 1 alkaloid, and 1 organic acid) were absent in all 12 AA samples, 9 metabolites were absent in all 12 S^l^S^l^ samples, but no metabolite was missing in all 4x samples (Supplementary Figure 3B). Of those 10 flavonoids absent in AA, 6 were glucoside and 1 was arabinoside (Supplementary Table 3). Our metabolome data indicated that 4x combined the metabolites of AA and S^l^S^l^ but did not produce any novel metabolite (Supplementary Figure 3B), as further detailed below.

### Effects of Allotetraploidization on Accumulation of Metabolites

**FIGURE 5:**
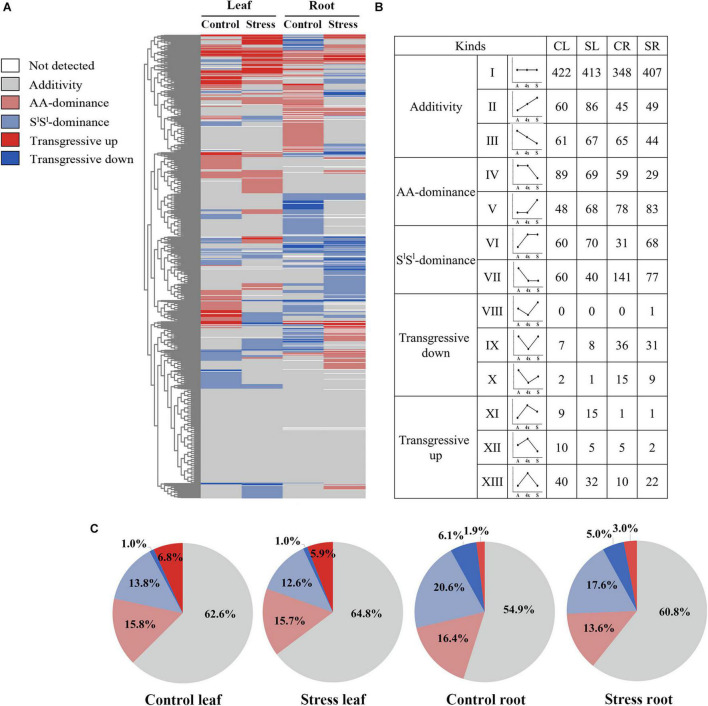
Summary of non-additive and additive accumulation of metabolite in the synthetic tetraploid wheat line under control and salinity stress condition. **(A)** Heat map showing non-additive and additive assignation of each detected metabolite; **(B)** number of metabolites of additive accumulation and each non-additive accumulation type; **(C)** the pie diagrams showing metabolite proportion among different non-additive accumulation types. The seedlings were exposed to 150 mM NaCl for 7 days. CL, control leaf; SL, stress leaf; CR, control root; SR, stress root.

We compared the relative concentrations of each metabolite in 4x and its two parents. According to effects of allotetraploidization on metabolite accumulation, we assigned all the 895 metabolites detected in 4x into 5 categories: (i) additive accumulation, (ii) *T. urartu*-parental dominant accumulation (AA-dominant), (iii) *A. longissima* parental dominant accumulation (S^l^S^l^-dominant), (iv) transgressive-up accumulation (T-up), and (v) transgressive-down accumulation (T-down) (Figure 5A and Supplementary Tables 4–7). If the concentration of a metabolite in 4x was similar to that of AA but was significantly different from that of S^l^S^l^, we assigned this metabolite as AA-dominant; the same definition applied for S^l^S^l^-dominant. If the concentration of a metabolite in 4x was higher than those of both parents (4x/AA > 2 VIP > 1 and 4x/S^l^S^l^ > 2 VIP > 1), the metabolite was assigned as T-up. Conversely, if the concentration of a metabolite in 4x was lower than those of both parents (4x/AA < 0.5 VIP > 1 and 4x/S^l^S^l^ < 0.5 VIP > 1), the metabolite was assigned as T-down.

**FIGURE 6:**
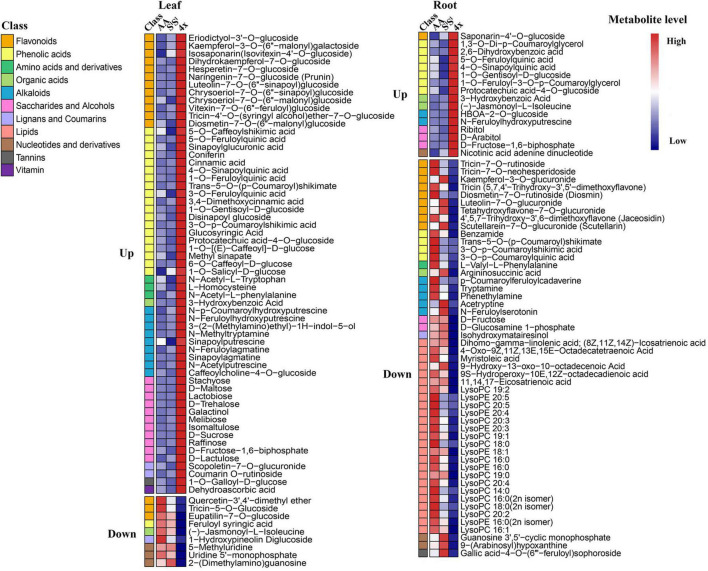
Heatmap showing metabolites with transgressive accumulation in a synthetic tetraploid under control condition. Transgressive up accumulation was defined as 4x/AA > 2 VIP > 1 and 4x/S^l^S^l^ > 2 VIP > 1, and transgressive down accumulation as 4x/AA < 0.5 VIP > 1 and 4x/S^l^S^l^ < 0.5 VIP > 1. The seedlings were exposed to 150 mM NaCl for 7 days. The first column showed class name of each metabolite, and the following three columns showed the relative levels of each metabolite for three wheat lines.

About 62.6% of all detected metabolites showed additive accumulation in control leaves, 64.8% in stressed leaves, 54.9% in control roots, and 60.8% in stressed roots of 4x. About 13.6–16.4% of all detected metabolites showed AA-dominant, 12.6–20.6% showed S^l^S^l^-dominant, and 6.9–8.0% showed transgressive accumulation (Figures 5B,C). Most key metabolites of primary metabolism pathways showed additive accumulation in both roots and leaves of 4x (Figure 4). We investigated the salinity stress-induced changing patterns of metabolite accumulation in 4x. Notably, in 4x leaves, 25 flavonoids showed changes from non-S^l^S^l^-dominant in the control to S^l^S^l^-dominant following salinity stress (Supplementary Figure 4). In particular, we focused on transgressive accumulation of metabolites in 4x (Figure 5). In control leaves, the number of metabolites showing T-up was greater than that showing T-down (Figure 6 and Supplementary Table 4). In contrast, in control roots of 4x, the number of metabolites showing T-up was less than that showing T-down. In control leaves of 4x, T-up was observed in 59 metabolites (19 phenolic acids, 12 flavonoids, 9 alkaloids, 11 carbohydrates, and 8 others), and very few metabolites showed T-down (Figure 6 and Supplementary Table 4). In control roots of 4x, T-down was observed in 51 metabolites (4 phenolic acids, 9 flavonoids, 5 alkaloids, 25 lipids, 2 carbohydrates, 2 nucleotide derivatives, and 4 others) (Figure 6 and Supplementary Table 6), and T-up was present in 16 metabolites (7 phenolic acids, 3 carbohydrates, and 2 organic acids, 1 flavonoid, 2 alkaloid, and 1 other) (Figure 6 and Supplementary Table 6). Together, the metabolic data indicated that, under control conditions, many carbohydrates showed T-up accumulation in 4x leaves, and many lipids showed T-down accumulation in 4x roots. This suggests that allotetraploidization strongly enhanced the accumulation of carbohydrates in leaves and reduced the accumulation of lipids in roots under normal conditions (Figure 6 and Supplementary Tables 4, 6).

### Salinity Stress Exacerbates Interspecific Differences in the Accumulation of Metabolites

**FIGURE 7:**
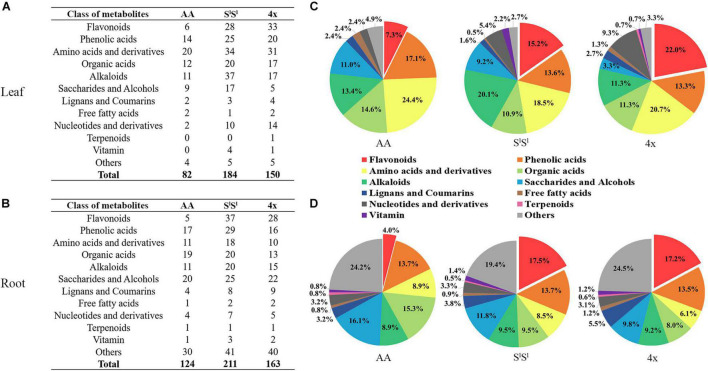
Salinity stress response of different types of metabolites in synthetic tetraploid wheat and its diploid parents. The number of metabolites with stress-enhanced accumulation in leaf **(A)** and root **(B)** was displayed, and the pie diagrams showed proportion among different metabolite types with enhanced accumulation in leaf **(C)** and root **(D)**. The seedlings were exposed to 150 mM NaCl for 7 days.

**FIGURE 8:**
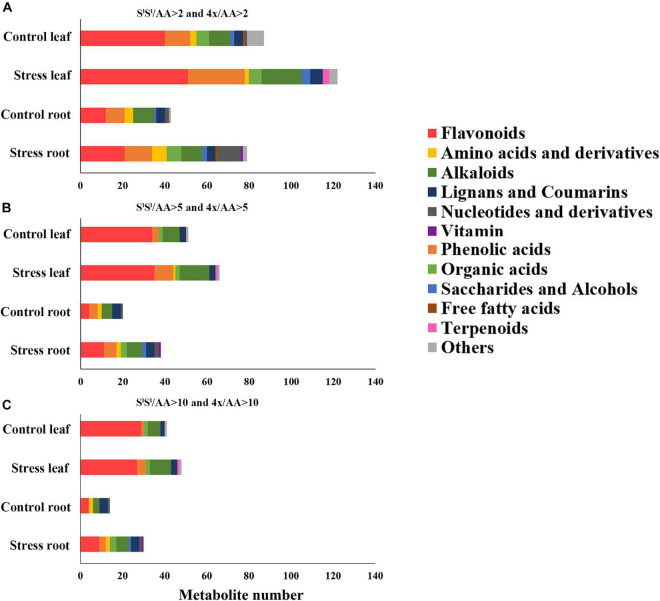
Comparison among three wheat lines in metabolite level for different types of metabolites. The metabolite was counted if its level in AA plants was lower than that in either of 4x and S^l^S^l^ plants. **(A)** The metabolite level in AA plants was lower twofolds than that in either of 4x and S^l^S^l^ plants (4x/AA > 2 VIP > 1 and S^l^S^l^/AA > 2 VIP > 1), **(B)** the metabolite level in AA plants was lower fivefolds than that in either of 4x and S^l^S^l^ plants (4x/AA > 5 VIP > 1 and S^l^S^l^/AA > 5 VIP > 1), and **(C)** the metabolite level in AA plants was lower 10-folds than that in either of 4x and S^l^S^l^ plants (4x/AA > 10 VIP > 1 and S^l^S^l^/AA > 10 VIP > 1). The seedlings were exposed to 150 mM NaCl for 7 days.

**FIGURE 9:**
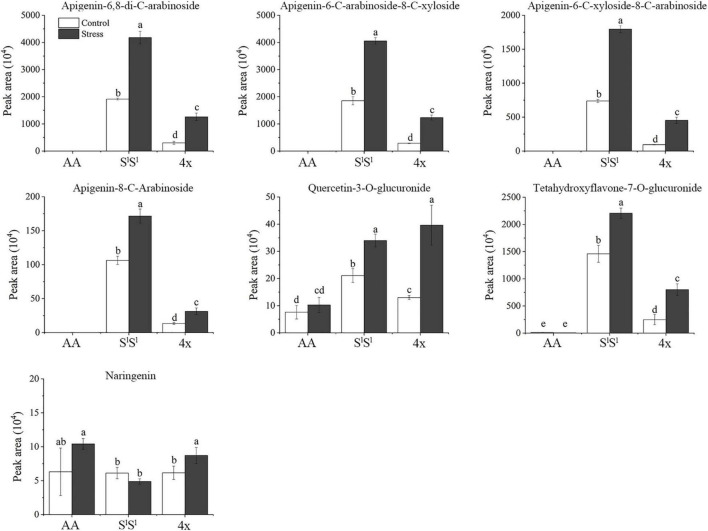
Examples of flavonoids showing distinct salinity stress response of *Triticum urartu* plants and the synthetic tetraploid wheat and *Aegilops longissima* plants. The seedlings were exposed to 150 mM NaCl for 7 days. The values are the mean (± SD) of three biological replicates. Different letters above the bar showed significant differences among wheat lines and treatments according to *t*-test (*P* < 0.05).

In leaves, salinity stress elevated the concentration of 82 metabolites in AA, 184 metabolites in S^l^S^l^, and 150 metabolites in 4x (Figure 7A and Supplementary Table 8). In roots, salinity stress enhanced the accumulation of 124 metabolites in AA, 211 metabolites in S^l^S^l^, and 163 metabolites in 4x (Figure 7B and Supplementary Table 9). In leaves, salinity stress elevated the accumulation of 28 flavonoids in S^l^S^l^, 33 flavonoids in 4x, and only 6 flavonoids in AA (Figures 7A,C). Similarly, in roots, salinity stress elevated the accumulation of 37 flavonoids in S^l^S^l^, 28 flavonoids in 4x, and only 5 flavonoids in AA (Figures 7B,D). To further explore the roles of flavonoid metabolism in salinity tolerance differences between AA and other wheat lines, we tabulated metabolites showing lower concentrations in AA than in 4x and S^l^S^l^ (4x/AA > 2 and S^l^S^l^/AA > 2) (Figure 8). We found 87 such metabolites (40 flavonoids) in control leaves, 122 (51 flavonoids) in stressed leaves, 43 (12 flavonoids) in control roots, and 79 (21 flavonoids) in stressed roots. We also tabulated the metabolites that displayed extremely low concentrations in AA compared with those in 4x and S^l^S^l^ (4x/AA > 10 and S^l^S^l^/AA > 10) and found 41 such metabolites (29 flavonoids, 6 alkaloids, and 6 others) in control leaves and 48 such metabolites (27 flavonoids, 10 alkaloids, 4 phenolic acids, and 7 others) in stressed leaves (Figure 8). Relative concentrations of some flavonoids with dramatic changes are shown in Figure 9. For these flavonoids, enhancement by salinity stress was much greater in leaves of 4x and S^l^S^l^ than in leaves of AA (Figure 9). Naringenin concentration was enhanced in 4x leaves but not in AA and S^l^S^l^ leaves (Figure 9). We also conducted a two-way ANOVA analysis (treatment vs. species) for each metabolite in which smaller *P*-value and greater F value indicated larger variation caused by species, stress, or their interaction (Supplementary Table 10). We listed the top 50 metabolites by ranking *P* values and F values of ANOVA analysis among species in leaves and found that 36 of the 50 top metabolites were flavonoids (Supplementary Table 10). In addition, principal component analysis (PCA) showed that the variation among the three wheat lines in flavonoid accumulation in roots was much smaller than that in leaves (Supplementary Figure 5). Collectively, our metabolomic data suggest that salinity stress exacerbated the intrinsic interspecific differences in flavonoid metabolism among the three wheat lines, the differences were much greater in leaves than in roots, and that the AA plants accumulated much lower levels of flavonoids than did S^l^S^l^ and 4x plants with the latter two lines being similar.

### Effects of Exogenous Flavonoid on Salinity Tolerance

**FIGURE 10:**
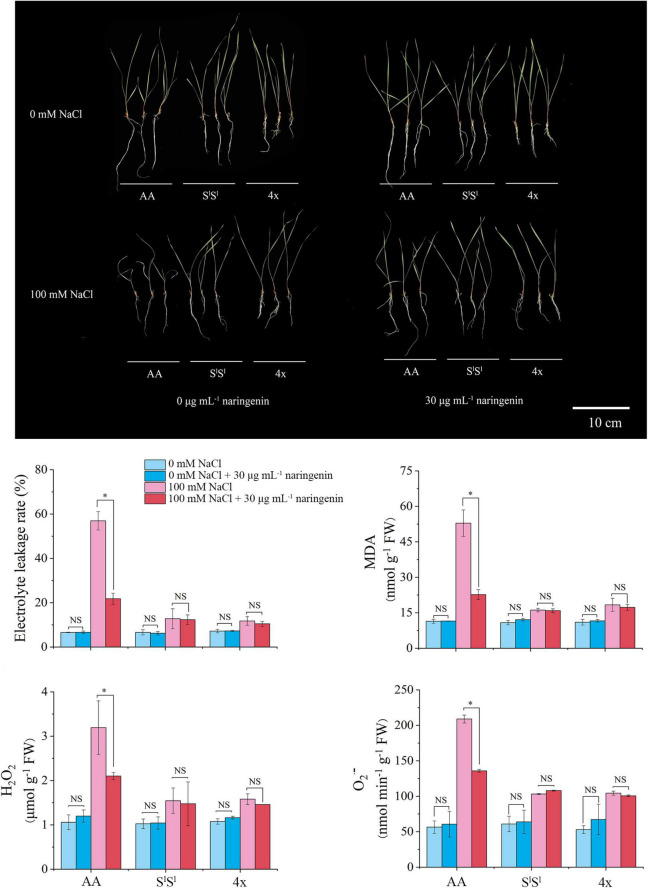
Alleviative effects of exogenous naringenin application on salinity stress-induced injury. The naringenin of 30 μg/mL was added into half-strength Hoagland nutrient solution with 100 mM NaCl or without NaCl. The seedlings were exposed to NaCl treatment for 10 days. The values are the mean (± SD) of three biological replicates. Star indicates significant differences between treatment with naringenin and treatment without naringenin and NS indicates no significant difference, according to *t*-test (*P* < 0.05).

We added the flavonoid (naringenin) to NaCl treatment solution. When stress and flavonoid treatments were applied for 10 days, we measured the MDA content, electrolyte extravasation rate, H_2_O_2_, and O_2_^⋅⁣–^ in leaves (Figure 10). Results showed that exogenous application of flavonoid significantly reduced the values of MDA, electrolyte extravasation rate, H_2_O_2_, and O_2_^⋅⁣–^ in salinity-stressed AA leaves, but not in leaves of 4x and S^l^S^l^ plants. These data revealed that exogenous flavonoid application to roots significantly relieved the injury of salinity stress on AA plants, but this alleviative effect was not observed in 4x and SS plants.

## Discussion

### S^l^S^l^ Subgenome Has Dominant Effect on Salinity Tolerance in Synthetic Allotetraploid Wheat (S^l^S^l^AA)

Plants in the whole biosphere collectively produce more than one million metabolites (Moghe et al., 2017). However, a single plant species may only produce 5,000–10,000 metabolites in different cell types and different development stages (Fernie et al., 2004; Fang et al., 2019), indicating dramatic inter-species differentiation (Buchanan et al., 2015). Primary metabolites and some key secondary metabolites are found in almost all plants as they play essential roles in growth, development, and environmental adaptation (Sulpice and McKeown, 2015; Moghe et al., 2017; Fang et al., 2019). However, a majority of secondary metabolites are only found in limited plant species or species groups (Moghe et al., 2017). It is therefore of considerable interest to understand the outcomes of metabolites following interspecific hybridization, alone or coupled, with whole-genome duplication (WGD), i.e., allopolyploidization. Here, we addressed this question using a stable synthetic allotetraploid (S^l^S^l^AA) and its two parental species, *Ae. longissima* (S^l^S^l^) and *T. urartu* (AA) (Zhang et al., 2013). Collectively, we detected 895 metabolites in these three lines under both normal and salinity stress conditions, totaling 36 samples. We found that each parental species lacked some metabolites, as expected, while the allotetraploid expressed all metabolites of both parents but did not produce any novel metabolite. This indicates that qualitatively, the synthetic allotetraploid manifested perfect metabolome additivity of its parents.

Quantitatively, however, only *ca.* 54.9–64.8% of metabolites showed strict (statistical) additive accumulation in the synthetic allotetraploid wheat (S^l^S^l^AA), while the rest showed either one parental dominance or transgressive accumulation. This is consistent with our earlier results demonstrating that there were extensive transgressive gene expressions in this allotetraploid (Zhang et al., 2016). Although most of the metabolites showed additive accumulation and 6.9–8.0% metabolites showed transgressive accumulation, the S^l^S^l^AA plants did not display transgressive salinity tolerance or mid-parent salinity tolerance level. Instead, the salinity tolerance of S^l^S^l^AA is highly similar to that of its S^l^S^l^ parent, suggesting that the S^l^S^l^ subgenome has a dominant role in this trait. Metabolome profiling of all three lines and the ANOVA analysis highlighted flavonoid metabolism as the most prominent interspecific differences. Ten flavonoids were absent in all 12 AA species samples but highly accumulated in S^l^S^l^ and S^l^S^l^AA plants. Also, S^l^S^l^ and S^l^S^l^AA plants showed similarly higher levels of many flavonoids under both control and stress conditions than AA plants.

### Synthetic Allotetraploid Wheat (S^l^S^l^AA) Acquired Strong Salinity Tolerance by Immediately Inheriting the Flavonoid Metabolism Traits of Its *A. longissima* Parent

Salinity stress exerts negative effects on plants *via* ion injury, osmotic stress, and reactive oxygen species (ROS) damage (Parida and Das, 2005; Gill and Tuteja, 2010; Wu et al., 2018; Yang and Guo, 2018; Flowers et al., 2019; Zhao et al., 2020). Previous studies have shown that lowering Na^+^ accumulation and ROS scavenging are major salinity tolerance mechanisms in wheat (Wei et al., 2013; Liu et al., 2014; Wang and Xia, 2018; Wang et al., 2020; Zheng et al., 2021). Elevated ROS levels will cause rapid plant death (Liebthal and Dietz, 2017), and enhancement of the capacity to detoxify ROS can significantly improve salinity tolerance of wheat plants (Liu et al., 2014; Wang and Xia, 2018; Wang et al., 2020; Zheng et al., 2021). In light of this, as AA was more saline-sensitive than S^l^S^l^ and S^l^S^l^AA, it was expected that Na^+^ concentration should be much higher in AA than in S^l^S^l^ and S^l^S^l^AA under salinity stress. However, our results showed that all three lines showed similar Na^+^ levels in both leaves and roots under salinity stress, indicating that salinity tolerance difference between AA and the other two lines was not attributable to differential Na^+^ accumulation. It is, therefore, more likely that the salinity tolerance differences between AA and the other two lines are attributable to differences in ROS metabolism.

Under salinity stress conditions, plants generally use antioxidant enzymes and non-enzymatic antioxidants, such as flavonoids, glutathione, and ascorbate, to scavenge excessive ROS. Severe stress frequently inactivates antioxidant enzymes, while flavonoids are extremely highly accumulated to play dominant roles in ROS scavenging (Agati et al., 2012). It was reported that enhanced flavonoid accumulation can improve the salinity tolerance of soybean plants (Bian et al., 2020). In this study, we found ROSs (H_2_O_2_ and O_2_^⋅⁣–^) concentrations were much higher in AA than in S^l^S^l^ and S^l^S^l^AA plants under salinity stress, while the three lines showed similar activities of antioxidant enzymes, such as SOD, POD, and CAT. Compared with S^l^S^l^ and S^l^S^l^AA, AA plants showed clear defects in the synthesis/accumulation of many flavonoids. Likewise, salinity stress enhanced the significant accumulation of many flavonoids in both roots and leaves in S^l^S^l^ and S^l^S^l^AA plants, while the accumulation of very few flavonoids was increased in AA plants. Exogenous flavonoid application reduced the ROS concentration in AA leaves but not in 4x and S^l^S^l^ leaves under the salinity stress condition. We propose that, under salinity stress, a lower accumulation of flavonoids may have led to a higher accumulation of ROS in AA plants, which provided a physiological explanation for its weaker salinity tolerance. Accordingly, we observed the alleviative effect of exogenous flavonoid application to salinity stress injury in AA plants but not in 4x and S^l^S^l^ plants. Combining this result with metabolome data, we conclude that flavonoids play a vital role in salinity tolerance differentiation between AA and other two wheat lines and that S^l^S^l^AA plants acquired strong salinity tolerance by immediately inheriting the flavonoid metabolism traits of its *A. longissima* parent.

Further studies are needed to elucidate the molecular basis underlying the metabolic characteristics of *A. longissima* (S^l^S^l^) which showed strong salinity tolerance and that is dominantly inherited to the synthetic allotetraploid wheat S^l^S^l^AA. If the salinity tolerance is controlled by a few genes of large effects, this species may have great potential to improve the salinity tolerance of hexaploid common wheat *via* genetic introgression or transgenic approaches.

## Materials and Methods

### Plant Materials

We used a synthetic tetraploid wheat line (accession AT2, genome S^l^S^l^AA, labeled as 4x) generated by crossing and chromosome-doubling of *Triticum urartu* (AA genome, labeled as AA) and *Aegilops longissima* (genome S^l^S^l^, labeled as S^l^S^l^). The initial seeds of AT2 were provided by Dr. Moshe Feldman from Weizmann Institute of Science, Israel, and the plants were then self-pollinated for six generations in our hands. We used euploid 4x plants as experimental materials. Seeds of 4x, AA, and S^l^S^l^ plants were sown in plastic pots containing washed sand. All the pots were watered with a half-strength Hoagland nutrient solution for 30 days. The 30-day-old seedlings (tillering stage) were treated with half-strength Hoagland nutrient solution containing 150 mM NaCl for 7 and 30 days, and control pots were watered with half-strength Hoagland nutrient solution for 7 and 30 days. All plants were grown in a greenhouse with a growth condition of 22–26°C day and 16–19°C night under 16-h light. When the seedlings were exposed to salinity stress for 7 days, metabonomic analysis and physiological measurements were conducted. Five plants for each wheat line were pooled as a biological replicate, with three biological replicates for all experiments. The plant samples were freeze-dried for metabonomic analysis and physiological measurements.

### Physiological Measurements

Photosynthetic parameters of fully expanded mature leaves were determined using a portable open flow gas exchange system LI-6800 (LI-COR, Lincoln, NE, United States). The photosynthetically active radiation was 1,200 μmol m^–2^ s^–1^. Chlorophyll contents in the leaves were determined according to the method of Ni et al. (2009). The activity of peroxidase (POD, EC 1.11.1.7) was measured according to the method of Muñoz-Muñoz et al. (2009). POD was extracted from fresh samples using cold extraction buffer (0.05 M phosphate buffer, pH 7.8). The reaction solution contains 5 μM 2-methoxyphenol and 0.01% H_2_O_2_ in 0.2 M phosphate buffer (pH 6.0). POD activity of the samples was measured by the addition of 40 μL crude enzyme extract to 3 mL reaction solution. An increase in absorbance at 470 nm was used to quantify the activity of POD, and one unit of POD was defined as an increase in OD value of 0.01 per minute. The activity of superoxide dismutase (SOD, EC 1.15.1.1) was determined by the SOD assay kit (Comin, Suzhou, China) using the NBT method (Tang, 1999), and one unit was defined as OD decrease per 30 min at 560 nm. The activity of catalase (CAT, EC 1.11.1.6) was measured using the method of Aebi (1984). CAT was extracted from fresh samples with 0.05 M phosphate buffer (pH 7.8). CAT activity was measured through the addition of 100 μL crude enzyme extract to a 3 mL reaction mix (0.05% H_2_O_2_ in 0.15 M phosphate buffer, pH 7.0). A decrease in the OD value at 240 nm was used to quantify catalase activity, and one unit of CAT was defined OD value decrease of 0.01 per minute. Total protein content was measured using the Coomassie Brilliant Blue G-250 staining method (Bradford, 1976). Activities of SOD, POD, and catalase were expressed as unit protein mg^–1^. Malondialdehyde (MDA) is an end product of lipid peroxidation. MDA concentrations of fresh samples were determined using the thiobarbituric acid reaction according to the method described in Tang (1999). The production rate of superoxide anion radical (O_2_^⋅⁣–^) in fresh samples was measured using the method described in Tang (1999). Concentrations of H_2_O_2_ in fresh samples were measured with an H_2_O_2_ assay kit (Sangon D799773-0050, Shanghai, China). The freeze-dried samples also were digested with 65% HNO_3_ at 120°C, and their Na^+^ and K^+^ concentrations were measured by an atomic absorption spectrophotometer (TAS-990super, PERSEE, China).

### Confocal Laser Scanning Microscopy Measurements

Germinated seeds for three wheat lines were grown in Petri dishes containing half-strength Hoagland nutrient solution for 2 days, and then all seedlings were transformed to buckets containing 2 L of half-strength Hoagland nutrient solution. After 10 days of hydroponic culture, the seedlings were exposed to 150 mM NaCl for 7 days, and control seedlings were grown with a half-strength Hoagland nutrient solution. CoroNa Green acetoxymethyl (AM) ester (product ID 2140298, Thermo Fisher Scientific, United States) was used to indicate the Na^+^ distribution in wheat root cells according to the method of Wang et al. (2016). CoroNa Green AM was dissolved in dimethyl sulfoxide as a stock solution. The roots were incubated in a dye-containing buffer solution (20 μM CoroNa Green AM, 5 mM KCl, 5 mM Ca^2+^-MES, pH 6.1) for 1 h in the dark. The dyed seedlings were washed with distilled water to remove CoroNa Green AM. The fluorescence intensity in the meristem and the maturation zone of the roots were measured using a confocal laser scanning microscopy with 488 nm excitation and 505–525 nm emission (Zeiss LSM880, Germany). Five individuals for each wheat line and each treatment were measured as five biological replicates. Microscopy image for each sample was analyzed with Zeiss ZEN 2.6 software (blue edition, Göttingen, Germany) to calculate fluorescence intensity for each root zone.

### Metabolic Profiling

We used a widely targeted metabolomics method to quantify metabolites using the workflow of Chen et al. (2013, 2020). Briefly, the freeze-dried leaves or roots were pulverized using a tissue lyser machine (MM 400, Retsch, Germany) at 30 Hz for 1.5 min, and then the metabolites of each pulverized sample were isolated using 1 mL of 70% methanol. Following centrifugation (12,000 rpm for 10 min), the extracted solutions were filtrated before analysis. To construct the MS2 spectral tag (MS2T) library, we loaded a mix of all the 36 extracts into a UPLC-MSMS system (QTRAP, ABSCIEX, United States) according to the workflow of Chen et al., 2013. Retention time, m/z, and fragmentation pattern of the detected metabolites were exposed to database MWDB-4.0 (MetWare Biological Science and Technology Ltd., Wuhan, China) to identify metabolites (Chen et al., 2013, 2020). Subsequently, the relative concentrations of 895 identified metabolites for each sample were quantified using the scheduled multiple reaction monitoring (MRM) method according to Chen et al., 2013. The variable importance in the projection (VIP) value of each variable in the orthogonal partial least-squares discriminant analysis (OPLS-DA) was used to discover differentially accumulated metabolite (DAM). We defined DAM between wheat lines or treatments as VIP > 1 and | Log2(fold change)| > 1.

### Exogenous Naringenin Treatment

Exogenous naringenin treatment was conducted according to the method of Novák et al. (2002). The germinated seeds were grown in a hydroponic condition supported by a half-strength Hoagland nutrient solution. Two-week-old seedlings were exposed to each treatment. Naringenin was dissolved in methanol as a stock solution (100 mg/mL) (Novák et al., 2002). The naringenin stock solution was added to half-strength Hoagland nutrient solution with 100 mM NaCl or without NaCl to a final concentration of 30 μg/mL. All the treatment solutions contained standard methanol of 0.03%. Each treatment solution was changed daily. When the treatments were applied for 10 days, we measured the MDA content, electrolyte extravasation rate, H_2_O_2_, and O_2_^⋅⁣–^ in mature leaves using the above method.

## Data Availability Statement

The original contributions presented in the study are included in the article/Supplementary Material, further inquiries can be directed to the corresponding author/s.

## Author Contributions

CY and BL conceived the study and designed experiments. TF, HL, XW, MT, CX, BX, and CY performed the experiment. TF, CY, BL, HL, XW, ZZ, RL, XG, and MT analyzed and interpreted the data. BL, CY, and TF drafted the article and carried out a critical revision of the article. All authors contributed to the article and approved the submitted version.

## Conflict of Interest

The authors declare that the research was conducted in the absence of any commercial or financial relationships that could be construed as a potential conflict of interest.

## Publisher’s Note

All claims expressed in this article are solely those of the authors and do not necessarily represent those of their affiliated organizations, or those of the publisher, the editors and the reviewers. Any product that may be evaluated in this article, or claim that may be made by its manufacturer, is not guaranteed or endorsed by the publisher.

## Supplementary Material

Supplementary Figure 1 Microgram showing Na^+^ distribution of a synthetic tetraploid wheat line and its diploid parents under control condition. Na^+^ distribution in root cell was indicated by CoroNa Green AM. The synthetic tetraploid wheat line (genome S^l^S^l^AA, labeled as 4x) was generated by crossing and chromosome-doubling of *Triticum urartu* (AA genome, labeled as AA) and *Aegilops longissima* (genome S^l^S^l^, labeled as S^l^S^l^).

Supplementary Figure 2 Relative Na^+^ concentration of a synthetic tetraploid wheat line and its diploid parents under control and salinity stress conditions. The values were expressed as fluorescence intensity from the microgram dyed by CoroNa Green AM. The values are the mean (± SD) of 5 biological replicates. Different letters above bar showed significant differences among different lines and treatments according to *t*-test (*P* < 0.05).

Supplementary Figure 3 Venn diagram showing comparison among a synthetic tetraploid wheat line and its diploid parents for detected metabolites. **(A)** The number of collectively detected metabolites; **(B)** Venn diagram showing comparison of number of collectively detected metabolites among three lines.

Supplementary Figure 4 Summary of non-additive and additive accumulation of flavonoid metabolite in the synthetic tetraploid wheat line under control and salinity stress condition.

Supplementary Figure 5 PCA plots showing all detected metabolites and all detected flavonoids under control (C) and salinity stress (S) condition.

Supplementary Table 1 Information of all detected metabolites.

Supplementary Table 2 Number of detected metabolites in each wheat line and each organ. CL, control leaf; SL, stress leaf; CR, control root; SR, stress root.

Supplementary Table 3 Relative concentration of the metabolites that were not detected in all AA plant samples. CL, control leaf; SL, stress leaf; CR, control root; SR, stress root.

Supplementary Table 4 Information of all transgressively accumulated metabolites in leaves of a synthetic tetraploid wheat line under control condition. Transgressive up accumulation: concentration of metabolite in 4x was higher than that of either of its parents (4x/AA > 2 VIP > 1 and 4x/S^l^S^l^ > 2 VIP > 1); transgressive down accumulation: concentration of metabolite in 4x was lower than that of either of its parents (4x/AA < 0.5 VIP > 1 and 4x/S^l^S^l^ < 0.5 VIP > 1).

Supplementary Table 5 Information of all transgressively accumulated metabolites in leaves of a synthetic tetraploid wheat line under salinity stress.

Supplementary Table 6 Information of all transgressively accumulated metabolites in roots of a synthetic tetraploid wheat line under control condition.

Supplementary Table 7 Information of all transgressively accumulated metabolites in roots of a synthetic tetraploid wheat line under salinity condition.

Supplementary Table 8 Fold changes (stress/control) of differentially accumulated metabolites in leaves of a synthetic tetraploid wheat line and its parents.

Supplementary Table 9 Fold changes (stress/control) of differentially accumulated metabolites in roots of a synthetic tetraploid wheat line and its parents.

Supplementary Table 10 Two-Way ANOVA analysis of treatment vs. species for the level of each metabolite. For each metabolite, smaller *P*-value and greater F value generated indicate larger variation caused by species, stress treatment, or their interaction.

## References

[B1] Aebi H. (1984). “Catalase *in vitro*” in *Methods in Enzymology.* ed. Packer L. (United States: Academic Press). 121–126. 10.1016/s0076-6879(84)05016-3 6727660

[B2] Agati G. Azzarello E. Pollastri S. Tattini M. (2012). Flavonoids as antioxidants in plants: location and functional significance. *Plant Sci.* 196 67–76. 10.1016/j.plantsci.2012.07.014 23017900

[B3] Banyai W. Sangthong R. Karaket N. Inthima P. Mii M. Supaibulwatana K. (2010). Overproduction of artemisinin in tetraploid Artemisia annua L. *Plant Biotechnol.* 27 427–433. 10.5511/plantbiotechnology.10.0726a

[B4] Bian X. Li W. Niu C. Wei W. Hu Y. Han J. (2020). A class B heat shock factor selected for during soybean domestication contributes to salt tolerance by promoting flavonoid biosynthesis. *New Phytol.* 225 268–283. 10.1111/nph.16104 31400247

[B5] Bradford M. M. (1976). A rapid and sensitive method for the quantitation of microgram quantities of protein utilizing the principle of protein-dye binding. *Anal. Biochem.* 72 248–254. 10.1016/0003-2697(76)90527-3 942051

[B6] Buchanan B. B. Gruissem W. Jones R. L. (2015). *Biochemistry and Molecular Biology of Plants.* West Sussex: John Wiley and Sons LTD. 1205.

[B7] Chen J. Hu X. Shi T. Yin H. Sun D. Hao Y. (2020). Metabolite-based genome-wide association study enables dissection of the flavonoid decoration pathway of wheat kernels. *Plant Biotechnol. J.* 18 1722–1735. 10.1111/pbi.13335 31930656 PMC7336285

[B8] Chen W. Gong L. Guo Z. Wang W. Zhang H. Liu X. (2013). A novel integrated method for large-scale detection, identification, and quantification of widely targeted metabolites: application in the study of rice metabolomics. *Mol. Plant* 6 1769–1780. 10.1093/mp/sst080 23702596

[B9] Chen Z. J. (2007). Genetic and epigenetic mechanisms for gene expression and phenotypic variation in plant polyploids. *Annu. Rev. Plant Biol.* 58 377–406. 10.1146/annurev.arplant.58.032806.103835 17280525 PMC1949485

[B10] Comai L. (2005). The advantages and disadvantages of being polyploid. *Nat. Rev. Genet.* 6 836–846. 10.1038/nrg1711 16304599

[B11] Dubcovsky J. Dvorak J. (2007). Genome plasticity a key factor in the success of polyploid wheat under domestication. *Science* 316 1862–1866. 10.1126/science.1143986 17600208 PMC4737438

[B12] Dvorak J. Akhunov E. D. (2005). Tempos of gene locus deletions and duplications and their relationship to recombination rate during diploid and polyploid evolution in the Aegilops-Triticum alliance. *Genetics* 171 323–332. 10.1534/genetics.105.041632 15996988 PMC1456522

[B13] Fang C. Fernie A. R. Luo J. (2019). Exploring the diversity of plant metabolism. *Trends Plant Sci.* 24 83–98. 10.1016/j.tplants.2018.09.006 30297176

[B14] Fernie A. R. Trethewey R. N. Krotzky A. J. Willmitzer L. (2004). Metabolite profiling: from diagnostics to systems biology. *Nat. Rev. Mol. Cell Biol.* 5 763–769. 10.1038/nrm1451 15340383

[B15] Firn R. D. Jones C. G. (2004). “The evolution of plant biochemistry and the implications for physiology” in *The Evolution of Plant Physiology.* eds Hemsley A. R. Poole I. (Oxford: Academic Press). 67–83.

[B16] Flowers T. J. Glenn E. P. Volkov V. (2019). Could vesicular transport of Na+ and Cl– be a feature of salt tolerance in halophytes. *Ann. Bot.* 123 1–18. 10.1093/aob/mcy164 30247507 PMC6344095

[B17] Fox D. T. Soltis D. E. Soltis P. S. Ashman T. L. Van de Peer Y. (2020). Polyploidy: a biological force from cells to ecosystems. *Trends Cell Biol.* 30 688–694. 10.1016/j.tcb.2020.06.006 32646579 PMC7484144

[B18] Gill S. S. Tuteja N. (2010). Reactive oxygen species and antioxidant machinery in abiotic stress tolerance in crop plants. *Plant Physiol. Biochem.* 48 909–930. 10.1016/j.plaphy.2010.08.016 20870416

[B19] Gornicki P. Zhu H. Wang J. Challa G. S. Zhang Z. Gill B. S. (2014). The chloroplast view of the evolution of polyploid wheat. *New Phytol.* 204 704–714. 10.1111/nph.12931 25059383

[B20] Huang S. Sirikhachornkit A. Su X. Faris J. Gill B. Haselkorn R. (2002). Genes encoding plastid acetyl-CoA carboxylase and 3-phosphoglycerate kinase of the Triticum/Aegilops complex and the evolutionary history of polyploid wheat. *Proc. Natl. Acad. Sci.* 99 8133–8138. 10.1073/pnas.072223799 12060759 PMC123033

[B21] Jiao Y. Wickett N. J. Ayyampalayam S. Chanderbali A. S. Landherr L. Ralph P. E. (2011). Ancestral polyploidy in seed plants and angiosperms. *Nature* 473 97–100. 10.1038/nature09916 21478875

[B22] Li G. Zhang T. Yu Z. Wang H. Yang E. Yang Z. (2021). An efficient Oligo-FISH painting system for revealing chromosome rearrangements and polyploidization in Triticeae. *Plant J.* 105 978–993. 10.1111/tpj.15081 33210785

[B23] Liebthal M. Dietz K. J. (2017). “The Fundamental Role of Reactive Oxygen Species in Plant Stress Response” in *Plant Stress Tolerance: methods and Protocols.* ed. Sunkar R. (New York: Springer). 23–39. 10.1007/978-1-4939-7136-7_2 28735389

[B24] Liu S. Liu S. Wang M. Wei T. Meng C. Wang M. (2014). A wheat similar to RCD-ONE gene enhances seedling growth and abiotic stress resistance by modulating redox homeostasis and maintaining genomic integrity. *Plant Cell* 26 164–180. 10.1105/tpc.113.118687 24443520 PMC3963566

[B25] Lou Y. Baldwin I. T. (2003). Manduca sexta recognition and resistance among allopolyploid Nicotiana host plants. *Proc. Natl. Acad. Sci.* 100 14581–14586. 10.1073/pnas.2135348100 14530394 PMC304122

[B26] Marcussen T. Sandve S. R. Heier L. Spannagl M. Pfeifer M. Jakobsen K. S. (2014). Ancient hybridizations among the ancestral genomes of bread wheat. *Science* 345:1250092. 10.1126/science.1250092 25035499

[B27] Moghe G. D. Leong B. J. Hurney S. M. Daniel Jones A. Last R. L. (2017). Evolutionary routes to biochemical innovation revealed by integrative analysis of a plant-defense related specialized metabolic pathway. *Elife* 6:e28468. 10.7554/eLife.28468 28853706 PMC5595436

[B28] Munns R. Tester M. (2008). Mechanisms of salinity tolerance. *Annu. Rev. Plant Biol.* 59 651–681. 10.1146/annurev.arplant.59.032607.092911 18444910

[B29] Muñoz-Muñoz J. L. García-Molina F. García-Ruiz P. A. Arribas E. Tudela J. García-Cánovas F. (2009). Enzymatic and chemical oxidation of trihydroxylated phenols. *Food Chem* 113 435–444. 10.1016/j.foodchem.2008.07.076

[B30] Ni Z. Kim E.-D. Ha M. Lackey E. Liu J. Zhang Y. (2009). Altered circadian rhythms regulate growth vigour in hybrids and allopolyploids. *Nature* 457 327–331. 10.1038/nature07523 19029881 PMC2679702

[B31] Novák K. Chovanec P. Škrdleta V. Kropáčová M. Lisá L. Němcová M. (2002). Effect of exogenous flavonoids on nodulation of pea (Pisum sativum L.). *J. Exp. Bot.* 53 1735–1745. 10.1093/jxb/erf016 12147723

[B32] Parida A. K. Das A. B. (2005). Salt tolerance and salinity effects on plants: a review. *Ecotox. Environ. Safe.* 60 324–349. 10.1016/j.ecoenv.2004.06.010 15590011

[B33] Pearse I. S. Krügel T. Baldwin I. T. (2006). Innovation in anti-herbivore defense systems during neopolypoloidy-the functional consequences of instantaneous speciation. *Plant J.* 47 196–210. 10.1111/j.1365-313X.2006.02776.x 16762034

[B34] Soltis P. S. Marchant D. B. Van de Peer Y. Soltis D. E. (2015). Polyploidy and genome evolution in plants. *Curr. Opin. Genet. Dev.* 35 119–125. 10.1016/j.gde.2015.11.003 26656231

[B35] Sulpice R. McKeown P. C. (2015). Moving toward a comprehensive map of central plant metabolism. *Annu. Rev. Plant Biol.* 66 187–210. 10.1146/annurev-arplant-043014-114720 25621519

[B36] Tang Z. (1999). *Experimental Guide for Plant Physiology.* Beijing: Science Press.

[B37] Uauy C. (2017). Plant genomics: unlocking the genome of wheat’s progenitor. *Curr. Biol.* 27 R1122–R1124. 10.1016/j.cub.2017.08.051 29065296

[B38] Van de Peer Y. Mizrachi E. Marchal K. (2017). The evolutionary significance of polyploidy. *Nat. Rev. Genet.* 18 411–424. 10.1038/nrg.2017.26 28502977

[B39] Wang F. Chen Z.-H. Liu X. Colmer T. D. Zhou M. Shabala S. (2016). Tissue-specific root ion profiling reveals essential roles of the CAX and ACA calcium transport systems in response to hypoxia in Arabidopsis. *J. Exp. Bot.* 67 3747–3762. 10.1093/jxb/erw034 26889007 PMC4896357

[B40] Wang M. Xia G. (2018). The landscape of molecular mechanisms for salt tolerance in wheat. *Crop J.* 6 42–47. 10.1016/j.cj.2017.09.002

[B41] Wang M. Yuan J. Qin L. Shi W. Xia G. Liu S. (2020). TaCYP81D5, one member in a wheat cytochrome P450 gene cluster, confers salinity tolerance via reactive oxygen species scavenging. *Plant Biotechnol. J.* 18 791–804. 10.1111/pbi.13247 31472082 PMC7004906

[B42] Wei D. Wang M. Xu F. Quan T. Peng K. Xiao L. (2013). Wheat oxophytodienoate reductase gene TaOPR1 confers salinity tolerance via enhancement of abscisic acid signaling and reactive oxygen species scavenging. *Plant Physiol.* 161 1217–1228. 10.1104/pp.112.211854 23321418 PMC3585591

[B43] Wu H. Shabala L. Azzarello E. Huang Y. Pandolfi C. Su N. (2018). Na+ extrusion from the cytosol and tissue-specific Na+ sequestration in roots confer differential salt stress tolerance between durum and bread wheat. *J. Exp. Bot.* 69 3987–4001. 10.1093/jxb/ery194 29897491 PMC6054258

[B44] Xing S. Guo X. Wang Q. Pan Q. Tian Y. Liu P. (2011). Induction and flow cytometry identification of tetraploids from seed-derived explants through colchicine treatments in *Catharanthus roseus* (L.) G. Don. *J. Biomed. Biotechnol.* 2011:793198. 10.1155/2011/793198 21660143 PMC3110335

[B45] Yang C. Zhao L. Zhang H. Yang Z. Wang H. Wen S. (2014). Evolution of physiological responses to salt stress in hexaploid wheat. *Proc. Natl. Acad. Sci.* 111 11882–11887. 10.1073/pnas.1412839111 25074914 PMC4136619

[B46] Yang Y. Guo Y. (2018). Elucidating the molecular mechanisms mediating plant salt-stress responses. *New Phytol.* 217 523–539. 10.1111/nph.14920 29205383

[B47] Yoo M. J. Liu X. Pires J. C. Soltis P. S. Soltis D. E. (2014). Nonadditive gene expression in polyploids. *Ann. Rev. Genet.* 48 485–517. 10.1146/annurev-genet-120213-092159 25421600

[B48] Zhang H. Bian Y. Gou X. Dong Y. Rustgi S. Zhang B. (2013). Intrinsic karyotype stability and gene copy number variations may have laid the foundation for tetraploid wheat formation. *Proc. Natl. Acad. Sci.* 110 19466–19471. 10.1073/pnas.1319598110 24218593 PMC3845155

[B49] Zhang H. Gou X. Zhang A. Wang X. Zhao N. Dong Y. (2016). Transcriptome shock invokes disruption of parental expression conserved genes in tetraploid wheat. *Sci. Rep.* 6:26363. 10.1038/srep26363 27198893 PMC4873831

[B50] Zhao C. Zhang H. Song C. Zhu J.-K. Shabala S. (2020). Mechanisms of plant responses and adaptation to soil salinity. *Innovation* 1:100017. 10.1016/j.xinn.2020.100017 34557705 PMC8454569

[B51] Zheng M. Lin J. Liu X. Chu W. Li J. Gao Y. (2021). Histone acetyltransferase TaHAG1 acts as a crucial regulator to strengthen salt tolerance of hexaploid wheat. *Plant Physiol.* 186 1951–1969. 10.1093/plphys/kiab187 33890670 PMC8331135

[B52] Zohary D. Feldman M. (1962). Hybridization between amphidiploids and the evolution of polyploids in the wheat (Aegilops-Triticum) group. *Evolution* 16 44–61. 10.2307/2406265

