# Supplementary figures and images for: Salinity Tolerance in a Synthetic Allotetraploid Wheat (SlSlAA) Is Similar to Its Higher Tolerant Parent Aegilops longissima (SlSl) and Linked to Flavonoids Metabolism
Source: Front Plant Sci. 2022 Mar 17;13:835498. doi: 10.3389/fpls.2022.835498 (PMC8968947; doi:10.3389/fpls.2022.835498)

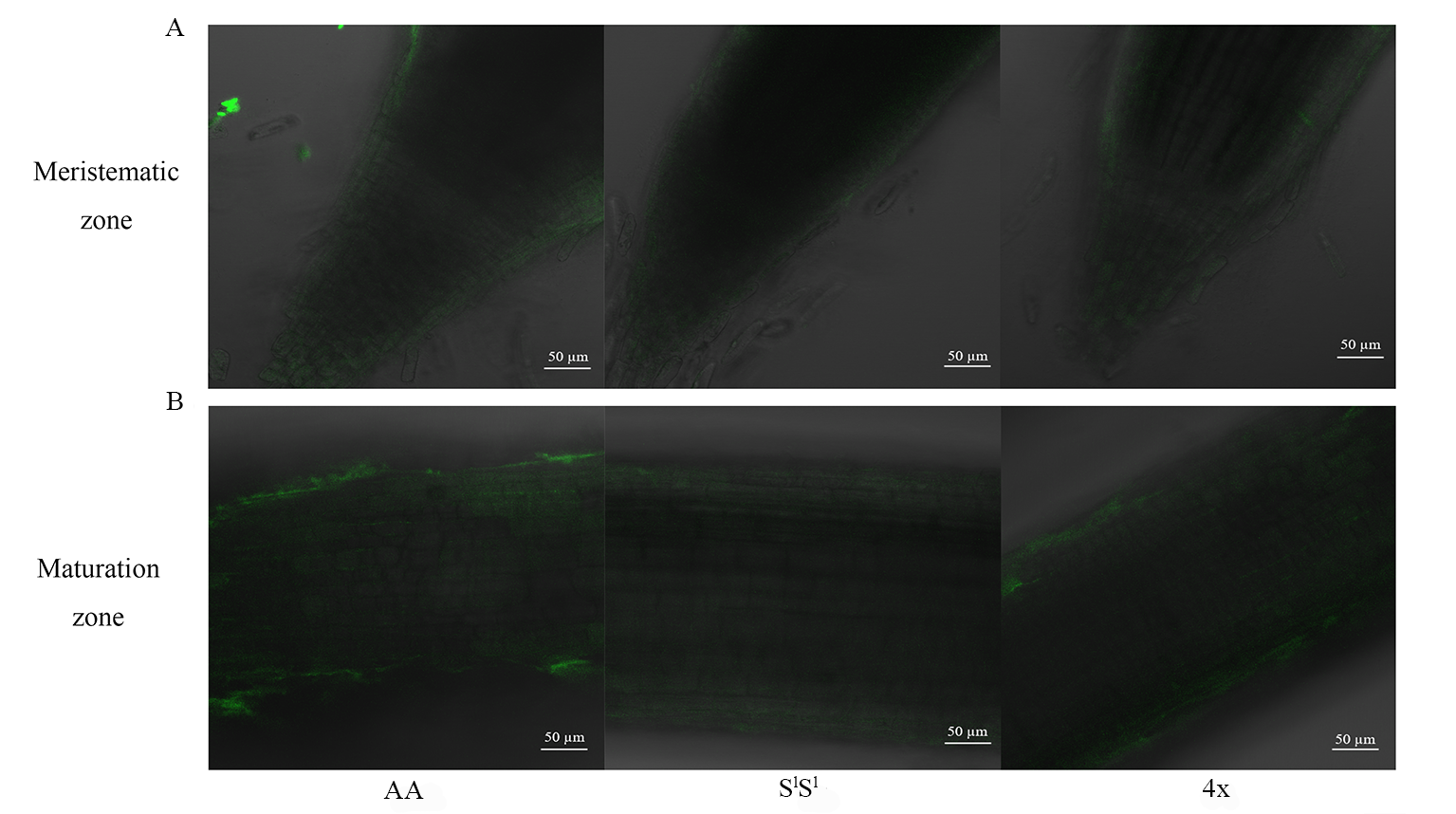

Supplement: Supplementary Figure 1 — Microgram showing Na+ distribution of a synthetic tetraploid wheat line and its diploid parents under control condition. Na+ distribution in root cell was indicated by CoroNa Green AM. The synthetic tetraploid wheat line (genome SlSlAA, labeled as 4x) was generated by crossing and chromosome-doubling of Triticum urartu (AA genome, labeled as AA) and Aegilops longissima (genome SlSl, labeled as SlSl). [file Image_1.TIF]

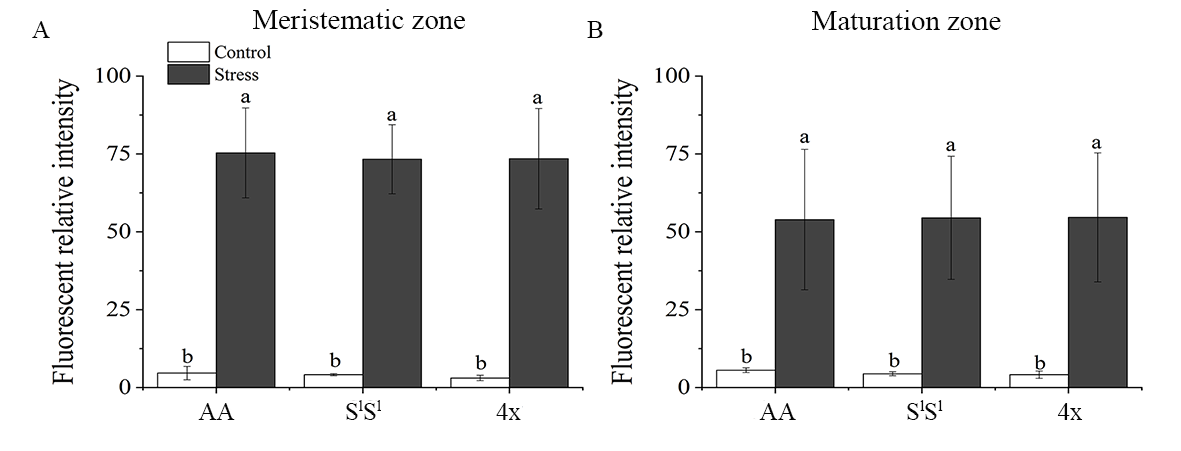

Supplement: Supplementary Figure 2 — Relative Na+ concentration of a synthetic tetraploid wheat line and its diploid parents under control and salinity stress conditions. The values were expressed as fluorescence intensity from the microgram dyed by CoroNa Green AM. The values are the mean (± SD) of 5 biological replicates. Different letters above bar showed significant differences among different lines and treatments according to t-test (P < 0.05). [file Image_2.TIF]

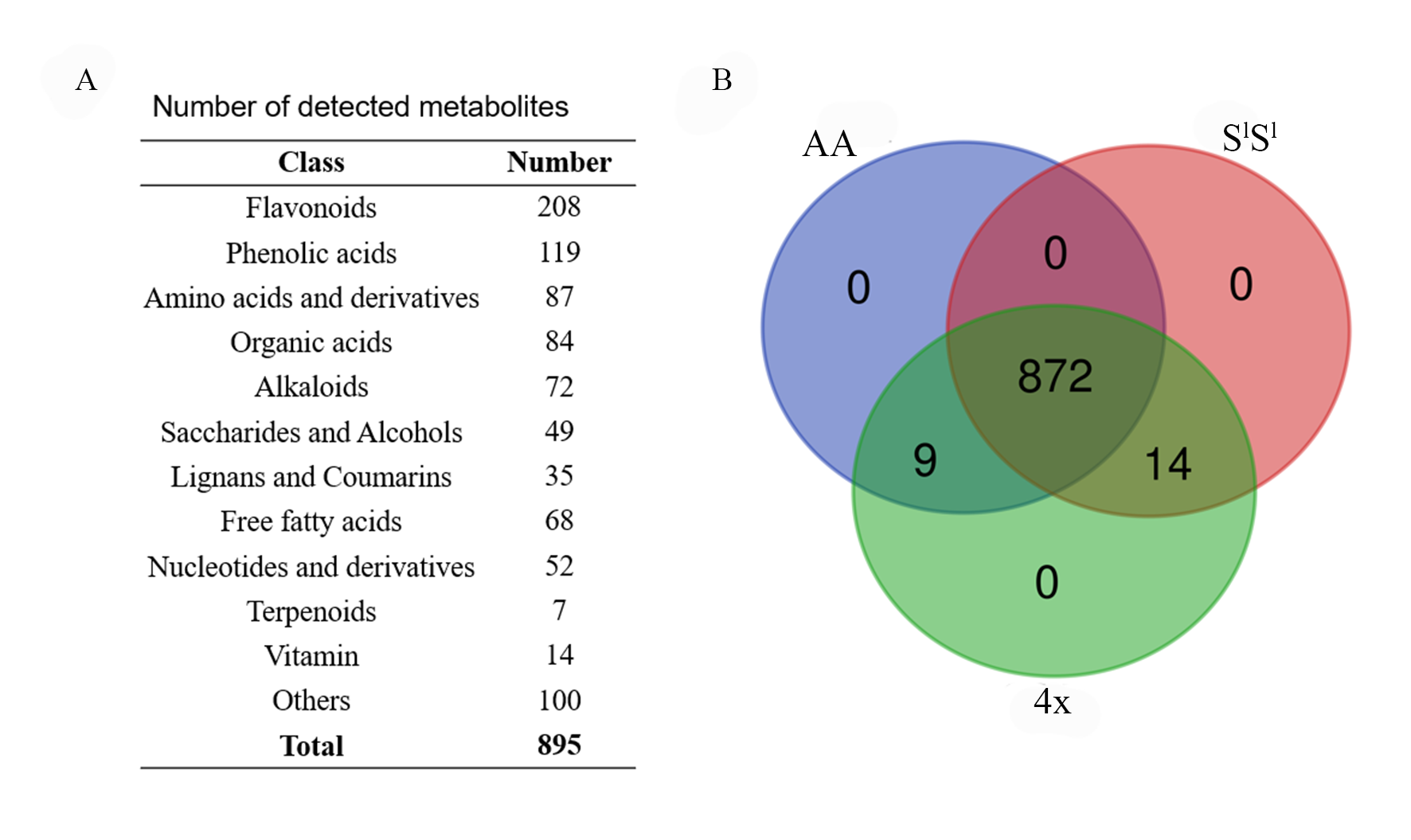

Supplement: Supplementary Figure 3 — Venn diagram showing comparison among a synthetic tetraploid wheat line and its diploid parents for detected metabolites. (A) The number of collectively detected metabolites; (B) Venn diagram showing comparison of number of collectively detected metabolites among three lines. [file Image_3.TIF]

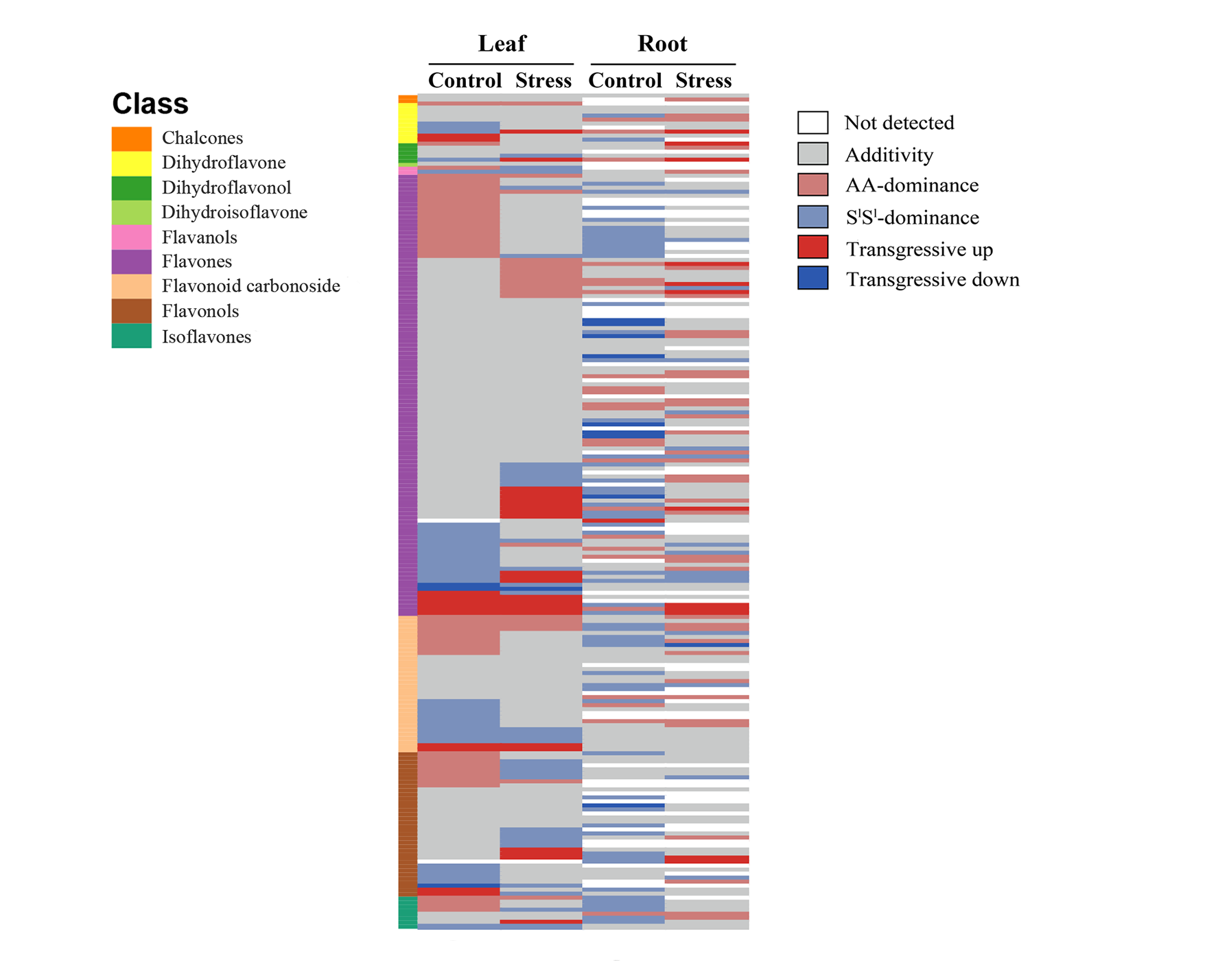

Supplement: Supplementary Figure 4 — Summary of non-additive and additive accumulation of flavonoid metabolite in the synthetic tetraploid wheat line under control and salinity stress condition. [file Image_4.TIF]

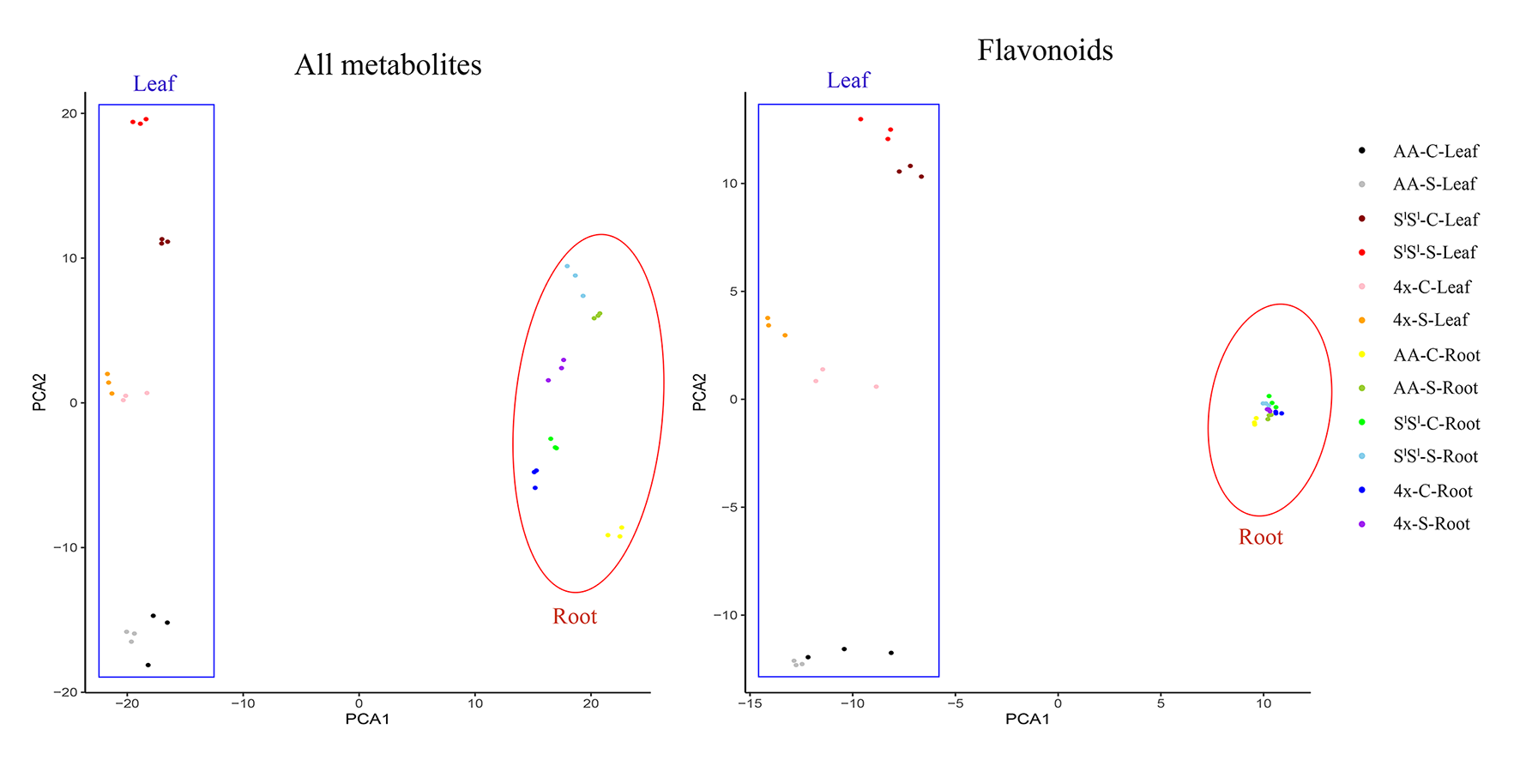

Supplement: Supplementary Figure 5 — PCA plots showing all detected metabolites and all detected flavonoids under control (C) and salinity stress (S) condition. [file Image_5.TIF]
